# Beyond the pandemic: physical activity and health behaviors as predictors of well-being among Filipino tertiary students

**DOI:** 10.3389/fpsyg.2025.1490437

**Published:** 2025-03-21

**Authors:** Lizamarie Campoamor-Olegario, Desiderio S. Camitan, Maria Luisa M. Guinto

**Affiliations:** ^1^College of Education University of the Philippines Diliman, Quezon City, Philippines; ^2^College of Human Kinetics, University of the Philippines Diliman, Quezon City, Philippines

**Keywords:** preventive health practices, active lifestyle promotion, higher education and well-being, adolescent and young adult health, holistic physical and mental well-being

## Abstract

**Introduction:**

This study investigates the relationship between positive health behaviors, physical activity, and well-being among 2,620 Filipino tertiary students, highlighting their enduring significance beyond the pandemic. While conducted in a post-quarantine context, the emphasis on the broader role of health behaviors in supporting student well-being, particularly amid academic pressures, mental health challenges, and sedentary lifestyles in an increasingly digital world, upholds the relevance of the study.

**Methods:**

Informed by the PERMAH model incorporating positive emotions, engagement, relationships, meaning, achievement and health in the investigation of well-being, the study employed multiple regression analysis to assess the impact of demographic variables, physical activity levels, and positive health behaviors on overall well-being. The predictor variables included physical activity, nutrition, relaxation, and preventive behaviors, with well-being as the outcome variable.

**Results:**

Findings revealed that the covariates of physical activity, nutrition, relaxation, and preventive behaviors moderately explained 30% of the total variability in post-pandemic well-being. Positive health behaviors significantly predicted the well-being components of positive emotions, engagement, meaning, accomplishment, and health scores. However, no significant associations were found among relationship, nutrition, and physical exercise scores.

**Conclusion:**

The results highlight the potential of positive health behaviors in shaping student well-being to address ongoing academic, mental health, and lifestyle challenges in tertiary education. The study underscores the need for holistic, evidence-based interventions integrating physical activity, nutrition, relaxation, and preventive behaviors into student support systems. While causality cannot be inferred, the findings assert the value of comprehensive health initiatives in fostering resilience and overall well-being. Future research is recommended to explore the interplay between nutrition, cognition, and mood, promote healthier campus environments, and develop targeted interventions for stress management and lifestyle improvement in academic settings.

## Introduction

Scholarly investigations have demonstrated the positive impact of physical activity (PA) on well-being (i.e., Belcher et al., 2021; Buecker et al., 2021; Marquez et al., 2020; Wright et al., 2021). Moreover, numerous studies have established associations between several positive health behaviors and well-being (Belcher et al., 2021), including proper nutrition (Firth et al., 2020; Muscaritoli, 2021; Wickham et al., 2020), adequate sleep (Ramar et al., 2021; Wickham et al., 2020), and regulated screen time (Onaolapo and Onaolapo, 2021; Oswald et al., 2020). Despite substantial evidence supporting these claims, reports document significant declines in physical activity and increases in sedentary behaviors among children and youth, raising concerns about the long-term consequences for their physical and mental health (Paterson et al., 2021; Runacres et al., 2021; Stockwell et al., 2021).

Before the pandemic, a disturbing trend of unhealthy behaviors was already observed among Filipino tertiary-level students. Acampado and Valenzuela (2018) reported that this demographic exhibited elevated levels of sedentary behavior, as evidenced by only 32.8% engaging in regular physical activity (PA). Sleep disturbances (Jorge II et al., 2020; Toyong, 2020) and nutritional deficiencies (Acampado and Valenzuela, 2018) among Filipino youth further compound these concerns. During the pandemic lockdown, this trend was amplified in the Philippines, as affirmed by Cruz et al. (2022), who found a significant decline in physical activity among university students, particularly females. This decline was coupled with negative impacts on various aspects of health, further highlighting the vulnerability of young people to the detrimental effects of restricted movement and social distancing measures on both physical and mental well-being. This research examines the complex interplay between physical activity, health behaviors, and well-being among Filipino tertiary students after the COVID-19 pandemic lockdown.

These pre-existing vulnerabilities, coupled with the COVID-19 pandemic’s disruptive impact on daily routines and access to resources, were further exacerbated by stringent lockdown measures in the Philippines (Inter-Agency Task Force for the Management of Emerging Infectious Diseases [IATF], 2020), intensifying poor health patterns, low PA levels, and suboptimal overall health. Studies reveal alarming statistics: 31% of Filipino college students reported insufficient sleep during the pandemic (University of the Philippines Population Institute, 2022), total PA levels among 1,042 Filipino students decreased by 6.84% (Cruz et al., 2022), and the country received a failing grade for youth physical activity with only 15.4% of Filipino youth engaging in moderate-to-vigorous physical activity per day (Cagas et al., 2022).

Research continues to emphasize the strong correlation between physical inactivity and poor mental health outcomes in young people, even as societies emerge from the pandemic. While Silva et al. (2020) observed alarming levels of anxiety, depression, and stress among young individuals reporting no physical activity during the pandemic, the implications extend beyond the lockdown period. A study by Li et al. (2022) reinforces that sedentary lifestyles and a lack of exercise remain significant risk factors for adverse mental health effects, even in the post-pandemic context. These findings suggest that the mental health challenges associated with inactivity during the pandemic may have lasting consequences. Furthermore, research by Chen et al. (2022) highlights that prolonged sitting was linked to poorer mental health, suggesting the pandemic might have amplified pre-existing vulnerabilities.

Studies indicate that college students continue to experience significant declines in physical and mental health post-pandemic (Moon and Lee, 2022; Nyunt et al., 2022), attributed to increased stress, academic disruptions, and financial challenges (Iglesia and Lu, 2021). These findings, combined with research showing the long-term psychological impact of pandemics (Solomou et al., 2024; Newnham et al., 2022), underscore the need for ongoing monitoring and intervention to address the evolving mental health needs of young adults, even as the physical threat of COVID-19 recedes.

### Culture and health behaviors

Filipino culture is characterized by collectivism, with family and community significantly influencing individual decisions, including those related to health and physical activity (Hofstede, 2001; Javier et al., 2022). From this cultural backdrop, young individuals are typically raised to consider family expectations and societal norms in their health choices. Such an environment can shape lifestyle choices, often placing health and well-being secondary to scholastic achievements. This strong emphasis on academic success, reinforced by parents and elders, frequently leads to students prioritizing good grades and deprioritizing extracurricular activities such as dance, sport, and exercise. Most Filipino families view education as vital to economic progress and, as such, implicitly discourage physical activity and sport participation if they are perceived as distractions to academic requirements (Medina and Guzman, 2015). This cultural norm may contribute to the widespread decline in physical activity among tertiary students, increasing the risk of sedentary lifestyles and related health concerns. Additionally, it normalizes sleep deprivation resulting from late-night study habits. The pressure to excel in school leads many students to sacrifice rest and sleep, adversely impacting cognitive function, emotional regulation, and overall well-being (Adelantado-Renau et al., 2019). Given the well-established association between insufficient sleep and poor mental health (Asghar et al., 2024; Tasnim et al., 2020; Wang and Bíró, 2021; Wilkes et al., 2021), this pattern poses significant challenges to student well-being.

Dietary habits among Filipino students are also heavily influenced by cultural practices, particularly family-oriented meal habits and food preferences. Traditional Filipino meals are often rich in carbohydrates, with rice serving as a staple food. Processed snacks are also commonly consumed, contributing to imbalanced nutritional intake (Serafico et al., 2023). While shared family meals strengthen social support, they can reinforce dietary habits that may not always align with optimal health recommendations.

University environments further shape student dietary behaviors. Many campus cafeterias offer limited affordable yet healthy food options, making it challenging for students to access nutritious meals (Suyu, 2016). This limitation often drives students toward cheaper, convenient, but less nutritious alternatives, such as instant noodles and deep-fried foods, widely available in and around academic institutions. The prevalence of street food culture compounds this issue, as inexpensive, high-fat, high-sodium snacks remain a popular choice among students facing financial constraints (Bondoc et al., 2019; Nonato et al., 2016).

These cultural and environmental factors could affect the health behaviors observed among Filipino tertiary students. Understanding these considerations is essential in designing effective interventions that promote healthier lifestyles while acknowledging cultural values. Addressing these issues requires a holistic approach, incorporating policy changes, educational initiatives, and institutional support to create environments that encourage balanced nutrition, adequate rest, and regular physical activity.

### The current study

This study aims to ascertain the predictors of well-being among Filipino tertiary students in the aftermath of the COVID-19 pandemic, focusing on the following variables: (1) demographic characteristics (sex, age, course cluster, and monthly household income); (2) positive health behavior (nutrition, PA, stress management, interpersonal relationships, and self-care); and (3) levels of PA.

While the well-being of individuals during the pandemic has been extensively studied, particularly in Western, educated, industrialized, rich, and democratic (WEIRD) societies (Andrada and Ozdemir, 2021; Shah et al., 2022; Thygesen et al., 2021), there is a notable gap in research on the immediate post-pandemic well-being of tertiary-level students in non-WEIRD settings like the Philippines. The study’s significance lies in its focus on the unique context of post-quarantine recovery in a country that implemented some of the world’s strictest and longest-lasting quarantine measures to contain the COVID-19 outbreak (Mathieu et al., 2020; See, 2021). Unlike many countries that had transitioned to in-person or hybrid teaching formats by 2021 (Balagtas, 2021), the Philippines only began reopening schools in August 2022, starting with primary and secondary levels (Agence France-Presse, 2022). Around the same time, tertiary institutions resumed in-person classes, predominantly through a flexible hybrid approach. Throughout the data-gathering phase of this study, the university adopted the blended learning model, integrating digital and face-to-face instruction to reduce the risk of a COVID-19 resurgence. This backdrop is essential to grasping the persistent ramifications of the pandemic for the Philippine education sector and the relevance of the country’s pandemic response to the study methodology and results.

Well-being is operationalized in this study within Seligman’s (2012) PERMA Model, which incorporates Positive Emotions, Engagement, Relationships, Meaning, and Achievement. This model is further expanded by including a physical health dimension, denoted by “H,” as proposed by Butler and Kern (2016). This additional health dimension updated the model with the PERMAH components, supported by 11 studies involving over 39,000 participants worldwide. The researchers combined a substantial pool of theoretically relevant questions about each of the six PERMAH domains to develop a comprehensive measure of well-being that includes stable attributes rather than transient mood states. Items exhibiting significant factor loadings and demonstrating satisfactory reliability were retained in the final measure, including the physical health dimension that assesses an individual’s capacity to undertake various daily activities effectively (Friedman and Kern, 2014).

Kern (2022) further outlined the approaches that support well-being in educational settings. To promote positive emotions, he emphasized the importance of encouraging positivity, minimizing negativity, and cultivating gratitude. Engagement is fostered by helping students recognize and utilize their strengths, practice mindfulness, and avoid distractions. Building relationships involves appreciating the strengths of others, developing friendships, and cultivating mutual respect and positive interactions. Finding meaning requires acknowledging significant life events—both fulfilling and challenging—as opportunities for growth, infusing tasks with purpose, and cultivating an appreciation of life. Accomplishment is facilitated by creating pathways for hope, tracking progress, and learning from setbacks. Finally, prioritizing health habits such as quality sleep, good nutrition, and regular physical activity is vital for overall well-being (Kern, 2022).

Investigating the relationship between positive health behaviors, such as physical activity (PA), and well-being is crucial to identify factors that promote holistic well-being in college students, especially in the challenging post-pandemic context (Rector et al., 2019; Lawton et al., 2017). This study contributes to a deeper understanding of the connection between physical and mental health, with practical implications for designing interventions to enhance the mental health of Filipino tertiary students (Maugeri et al., 2020). By examining the role of positive health behaviors, including PA levels, on well-being, this research highlights practical strategies for fostering mental health amidst adversity. It also aligns with the Sustainable Development Goal (SDG) of promoting healthy lifestyles. Understanding these relationships empowers the development of tailored programs supporting physical and mental health, potentially mitigating the pandemic’s adverse consequences on this vulnerable population. Following this rationale, we hypothesize that:

*H_0_1*: Physical activity levels predict well-being among Filipino tertiary students.

*H_0_2*: Dimensions of positive health behaviors predict well-being among Filipino tertiary students.

## Methodology

### Research design

The present study uses a cross-sectional design involving multiple regression models to test the assumption that various dimensions of PA and engagement in positive health behaviors (as operationalized by the PERMAH elements) are predictors of well-being. Through multiple regression analysis, the study examines the associations between each dimension of PA and the individual components of PERMAH.

### Research participants

A total of 2,620 college students from a university system with eight constituent universities located across the three major islands of the Philippines were enlisted to respond to an online survey (see Table 1). The university draws in students from various backgrounds and academic interests, reflecting the diverse demographic of Filipino tertiary students. Participant selection was conducted through stratified cluster sampling. First, the university’s seven major campuses were identified, representing both urban and rural areas to capture the diversity of the student body. Each campus was treated as a separate and independent cluster, meaning the sampling process was conducted entirely within each campus, ensuring representation from all geographical locations. Second, a specific number of Physical Education (PE) classes were randomly chosen from each campus cluster. PE classes were chosen as they are mandatory general education courses, thus ensuring a diverse sample of students from various fields of study. Third, PE teachers from the selected classes were then involved in the recruitment process, inviting their students to participate in the study. This approach generated a sample that represents the overall composition of the student population.

**Table 1:** Profile of the participants.

Participant characteristic	*n*	%
Sex at birth^a^		
Female	1,528	58
Male	1,090	42
Age^a^		
17–19	1,565	59.7
20–22	925	35.3
23–25	120	4.6
26 years old and older	10	0.4
Course cluster		
Science and Technology	1,598	61
Arts and Letters	415	16
Social Science and Law	371	14
Management and Economics	239	9
Monthly family income (in PHP)^a^		
0–12,000	225	9
13,00–24,000	407	16
25,00–48,000	583	22
49,000–84,000	475	18
85,00–132,000	372	14
133,00–228,00	262	10
229,000 and higher	254	10
No regular income	3	0
Not applicable	10	0
Do not know	13	0
Refused to answer	15	1

^a Data from one participant is missing. b x̄ age = 19.59; median age = 19; SD = 1.65.^

Table 1 reflects the broad and diverse sample, with 1,090 indicating their sex as male and 1,528 as female. The age range spanned from 17 to 34, with a mean age of 19.59 (SD = 1.65). The majority of the students (61%) were enrolled in the science and technology cluster of courses, the rest in the arts and letters cluster (16%), the social sciences and law cluster (14%), and the management and economics cluster (9%).

### Inclusion criteria

The following criteria were used in choosing whom to include in the study: Firstly, they must be tertiary students. Secondly, they must have been enrolled in the first semester of the academic year (AY) 2022–2023 at any of the seven campuses of the university system: Campus 1 (*n* = 1,681), Campus 2 (*n* = 65), Campus 3 (*n* = 332), Campus 4 (*n* = 186), Campus 5 (*n* = 53), Campus 6 (*n* = 185), and Campus 7 (*n* = 116). Lastly, participants had to be citizens of the Philippines. Individuals below 17 or above 34, those who declined to provide complete responses to the online survey questionnaires, or those who did not reside in the Philippines were excluded from the study.

### Research instruments

The study utilized Google Forms to gather sociodemographic data and dispatch the self-administered scales to measure key variables. It employed three main instruments: the International Physical Activity Questionnaire (IPAQ) to measure the participants’ PA levels, the Positive Health Behavior Scale (PHBS) to assess their engagement with positive health behaviors, and the PERMA(H) Profiler to gauge their overall well-being.

The IPAQ, a self-report scale for adults aged 18–65, measures PA in work, transportation, domestic/gardening, and leisure (Craig et al., 2003). It categorizes respondents into insufficiently active, moderately active, and health-enhancing PA. Despite documented reliability (*α* = 0.81) and validity from studies like that of Lee et al. (2011) and Malete et al. (2022) and a systematic review by Prince et al. (2008), some studies report minimal concordance with other PA measures, particularly in the work domain (Cleland et al., 2018).

The PHBS, developed by Woynarowska-Sołdan and Węziak-Białowolska (2012), assesses four domains of positive health behaviors: nutrition, PA, relaxation/mental health, and preventive behaviors. It provides individual domain and aggregate scores, with higher scores indicating stronger adherence. Psychometric evaluations confirm its reliability (α = 0.76–0.90) and validity (Ghiasvand et al., 2020; Hildt-Ciupińska and Pawłowska-Cyprysiak, 2020; Kong et al., 2023) across populations.

The PERMA(H) Profiler, also known as the PERMAH Well-being Survey (McQuaid, 2023), measures well-being across positive emotion, engagement, relationships, meaning, achievement, and health using a 10-point Likert scale (Butler and Kern, 2016). Higher scores indicate greater well-being. It has demonstrated adequate psychometric properties (*α* = 0.71–0.94) and sufficient reliability (Cobo-Rendón et al., 2020; Butler and Kern, 2016).

The PERMA(H) Profiler has also been used in studies involving Filipino participants. For instance, Camitan and Bajin (2021) observed “excellent” internal consistency (*α* = 0.92) among 533 adult Filipinos during the COVID-19 pandemic. Similarly, the scale has been observed to have “good” (*α* = 0.81; *ω =* 0.88) reliability in research consisting of 146 Filipino preservice teachers (Camitan IV and Campoamor-Olegario, 2023). In addition, Moog (2021) used the PERMA(H) Profiler to measure well-being among 117 Filipino graduate students, finding adequate internal consistency. Reyes et al. (2021) found that the PERMA(H) Profiler has “good” internal consistency among 339 Filipinos nearing retirement age. Lastly, the study of Villarino et al. (2023) showed Cronbach alpha values of the PERMA(H) factor ranging from 0.60 to 0.95 among 178 rural college students in the Philippines.

While the PERMA(H) Profiler, the PHBS, and IPAQ demonstrated reliability and validity across various studies, it is essential to acknowledge the inherent limitations of self-reported data. Recall bias may influence participants, who are challenged to remember past behaviors accurately. They may also be affected by social desirability bias, which may lead them to provide responses they consider to be more socially acceptable rather than what is truthful. To address these concerns, the questionnaire included clear and concise instructions to guide participants in responding as accurately as possible while assuring them of privacy, confidentiality, and anonymity, thereby reducing potential fear of judgment or repercussions. Additionally, instruments were pilot-tested with a separate group of tertiary students to refine the wording of questions, ensuring clarity and comprehension, and minimizing misinterpretations. A pre-recorded video detailing the study’s purpose and the importance of honest and thoughtful responses was also presented to participants. Nonetheless, self-reporting remains subject to individual interpretation and response tendencies, so caution should be considered when analyzing the data.

In the current study (*n* = 2,620), both the PERMA(H) Profiler (*α* = 0.93; *ω* = 0.94) and PHBS (*α* = 0.81; ω = 0.82) demonstrated “excellent” and “good” internal consistency, respectively, indicating reliability in measuring the current sample’s positive health behaviors and well-being. IPAQ (*α* = 0.8162; *ω =* 0.63) exhibited “questionable” internal consistency in this study, suggesting caution in interpreting IPAQ scores. It should be noted that the PERMA Profiler for this investigation includes the component of health; thus, adopting the acronym PERMAH throughout the report affirms health as one of the essential components of well-being.

### Ethical considerations

This study prioritized respect for the participants’ privacy, confidentiality, and anonymity. Participants were assured that they could withdraw from the study at any time without consequence. Before participating, they received detailed information about the study and were required to provide informed consent. To proceed with the survey, they were requested to tick a consent box on the first page of the online questionnaire. No incentives were offered in exchange for their participation.

Rigorous measures were implemented to prevent privacy breaches, maintain anonymity, and protect data security during the online questionnaire administration, fostering a sense of safety and protecting participant contributions to the research. Moreover, all research-related communications were conducted with transparency and respect. Ultimately, the well-being of research participants was safeguarded, ensuring no harm was inflicted during the study. Institutional ethical clearance was granted under Ethics Review Board Reference Number RGAO-2022-0760.

### Data gathering procedure

Although the nationwide community quarantine had been lifted during the data collection, schools and universities implemented a hybrid learning model, with classes primarily conducted online or remotely and limited face-to-face meetings. Data collection took place from November to December 2022, with the researchers collaborating with the PE department heads from various university campuses in different parts of the country. The PE teachers from different campuses invited tertiary students to participate in this study.

Prior to data gathering, the research instruments were pilot-tested with tertiary students from another school to ensure clarity and reliability. To begin the data-gathering process, the researchers presented a prerecorded video explaining the nature of the investigation and providing instructions for completing the measurement scales. Participants were then asked to complete a consent form before responding to the online questionnaires in Google Forms, which took approximately 20 min to answer.

The first section of the Google Form featured the study’s title, a brief overview of the research project, and a consent statement. Then, socio-demographic information and responses to the IPAQ, PHBS, and PERMA Profiler were collected. Afterwards, participants were debriefed on the research objectives and methodology. If participants chose not to provide consent, they were instructed to exit the Google Form site, and any previously submitted information was automatically discarded. Subsequently, data collected from these forms were downloaded and exported to IBM’s Statistical Package for the Social Sciences (SPSS) for analysis.

### Data analysis

A multiple regression analysis was performed to assess how different dimensions of positive health behaviors, including PA levels, predict the well-being of Filipino undergraduate students. This analytical approach treated PA levels and positive health behavior dimensions as predictor variables, while well-being elements served as outcome variables. By employing multiple regression, the study was able to isolate the distinct contribution of PA levels to well-being while controlling for other pertinent factors, such as age, sex, and combined monthly income. Regression coefficients and *p*-values were computed to ascertain which PA dimensions are most predictive of each element of well-being within the PERMAH framework.

## Results

Table 2 presents the PERMAH profile of university students after the lockdown was lifted. The results indicate that students experienced normal functioning in engagement and relationships. However, they exhibited suboptimal functioning in all other PERMAH elements, including positive emotions, meaning, achievement, negative emotions, health, loneliness, happiness, and overall well-being.

**Table 2:** PERMAH profile of university students during the COVID-19 pandemic.

PERMAH element	x̄	SD	Interpretation
Positive emotions	6.15	1.80	Sub-optimal functioning
Engagement	7.07	1.46	Normal functioning
Relationships	6.92	1.83	Normal functioning
Meaning	6.29	1.95	Sub-optimal functioning
Accomplishment	6.27	1.62	Sub-optimal functioning
Negative emotions	5.90	1.76	Sub-optimal functioning
Health	5.60	1.99	Sub-optimal functioning
Loneliness	5.51	2.47	Sub-optimal functioning
Happiness	6.43	1.96	Sub-optimal functioning
Overall well-being	6.53	1.45	Sub-optimal functioning

PERMAH, positive emotion, engagement, relationships, meaning, accomplishment, and health.

Table 3 presents the distribution of PA levels among the participants, as classified by the IPAQ. Fifty per cent of participants were categorized as moderately active, followed by 28% who met the criteria for health-promoting PA and 23% who were classified as insufficiently active.

**Table 3:** Distribution of physical activity levels among university students after the COVID-19 pandemic.

IPAQ categories	*n*	%
Health promoting PA	533	28%
Moderately active	948	50%
Insufficiently active	432	23%

IPAQ, international physical activity questionnaire.

Table 4 paints a complex portrait of health behavior adherence post-lockdown, with the subscales providing a more nuanced understanding. While the domain of preventive behaviors (*M* = 1.96, SD = 0.97) demonstrates an overall positive trajectory, it is not without nuances. The high adherence to brushing teeth twice daily (*M* = 2.56, SD = 0.72) and following doctor’s recommendations (*M* = 2.45, SD = 0.81) underscores a proactive approach to basic health maintenance and responsible healthcare utilization. However, the lower scores for dental check-ups (*M* = 1.35, SD = 1.14) and vaccinations (*M* = 1.89, SD = 1.06) suggest a potential gap in preventive care seeking and adherence to recommended health schedules.

**Table 4:** Mean and standard deviation of PHBS after lifting COVID-19 lockdowns.

PHBS item	x̄	SD
Preventive behaviors	1.96	0.97
I brush my teeth at least twice a day.	2.56	0.7
If I get sick and have a doctor’s appointment, I follow the doctor’s recommendations.	2.45	0.8
I avoid excessive sunbathing.	2.13	1.0
I have a flu vaccine, according to recommendations.	1.89	1.1
I have a dental check-up every 6 months.	1.35	1.1
I measure my blood pressure once a year.	1.35	1.1
Relaxation and behaviors related to mental health	1.61	0.86
I spend at least 20–30 min a day resting/ relaxing.	2.27	0.8
I spend time with colleagues/ friends at least once a month.	2.21	0.8
I ask other people for help in difficult situations.	1.65	0.9
I am positive about myself and the world.	1.56	0.8
I get at least 6–7 h of sleep every night.	1.41	1.0
I cope well with stress.	1.37	0.8
I go to bed at regular hours.	0.81	0.9
Nutrition	1.38	0.91
I have at least 3 meals a day with a regular meal pattern.	1.97	0.9
I have breakfast at home every morning (more than a glass of milk, tea, coffee, or other beverage).	1.63	1.1
I eat vegetables at least once a day.	1.60	0.9
I limit the amount of sweets	1.45	0.9
I limit the intake of animal fats.	1.28	0.9
I limit the intake of salt.	1.24	0.9
I eat fruit at least once a day.	1.21	0.8
I avoid snacking between meals (e.g., between lunch and a light afternoon meal).	1.18	0.9
I drink at least 2 glasses of milk, kefir, or yoghurt daily.	0.86	0.9
Physical activity	1.21	0.9
I increase physical activity and physical effort in everyday life.	1.64	0.9
I exercise daily for at least 30 min with moderate or vigorous intensity.	1.20	0.9
I do strength-building exercises for my main muscle groups at least twice a week.	1.15	1.0
I limit my recreational screen time.	0.86	0.8
Total	1.54	0.91

Response options: 0 = never or almost never, 1 = sometimes, 2 = often, 3 = always or almost always.

Relaxation and mental health (*M* = 1.61, SD = 0.86) illustrate resilience and vulnerability. The relatively high scores for resting and relaxing (*M* = 2.27, SD = 0.86) and socializing (*M* = 2.21, SD = 0.89) suggest an awareness of the importance of self-care and social connection for mental well-being. Nonetheless, the struggles with sleep regularity (*M* = 0.81, SD = 0.90), stress coping (*M* = 1.37, SD = 0.95), and adequate sleep duration (*M* = 1.41, SD = 1.02) point towards the lingering impacts of the pandemic or perhaps pre-existing challenges that were exacerbated during lockdowns.

Nutrition (*M* = 1.38, SD = 0.91) reveals a mix of dietary habits. The relatively high frequency of having at least three meals a day (*M* = 1.97, SD = 0.95) suggests a foundation of regular eating patterns. However, the lower scores for limiting fats (*M* = 1.28, SD = 0.93), salt (*M* = 1.24, SD = 0.92), and snacking (*M* = 1.18, SD = 0.92), along with infrequent dairy consumption (*M* = 0.86, SD = 0.92), point toward areas for improvement in dietary quality and balance. These findings might reflect challenges such as limited access to or affordability of healthy food options, a lack of adequate nutritional knowledge, or the influence of ingrained dietary habits and cultural preferences.

The lowest mean score of physical activity among the subscales (*M* = 1.21, SD = 0.9) reveals a concerning lack of regular PA among the participants. While there is some effort to incorporate activity in daily life (*M* = 1.64, SD = 0.93), the relatively low scores for strength training (*M* = 1.15, SD = 1.02) and limiting recreational screen time (*M* = 0.86, SD = 0.81) underscore areas needing significant improvement. These findings reflect limited access to fitness facilities, a lack of awareness about the benefits of exercise, or the pervasive influence of sedentary lifestyles and digital distractions.

Along with IPAQ categories and PHBS scores, the demographic variables of the participants were used as predictors of PERMAH elements in seven multiple regression models, one for each PERMAH element, one for overall well-being, and another for health as outcome variables. Table 5 summarizes the adjusted R^2^ of the aforementioned multiple regression models.

**Table 5:** Adjusted *R*^2^ of each regression equation.

	O	P	E	R	M	A	H
Adjusted *R*^2^	0.30	0.28	0.15	0.16	0.25	0.23	0.30

O, overall well-being; P, positive emotion; E, engagement; R, relationships; M, meaning; A, accomplishment; H, health.

Table 5 illustrates that the covariates employed as regressors collectively account for 30% of the total variance in the participants’ overall well-being scores. Specifically, they contribute to 28% of positive emotion scores, 15% of engagement scores, 16% of positive relationship scores, 25% of meaning scores, 23% of accomplishment scores, and 30% of health scores. These negligible adjusted *R*^2^ values suggest that the covariates in each model do not explain a substantial portion of the variability in PERMAH scores. However, these results indicate that while the covariates used in the regression models demonstrate minimal explanatory power for the observed variation in the PERMAH scores, their impact on overall well-being was more substantial, as evidenced by an adjusted *R*^2^ of 0.30. This finding implies that the covariates have moderate explanatory power for overall well-being.

### Demographic variables as predictors of PERMAH

Table 6 summarizes the results of seven multiple regression models focusing on demographic variables as regression coefficients for PERMAH and overall well-being. As shown in Table 6, age was significantly correlated with overall well-being (*p* = 0.02, *β* = −0.05), positive emotions (*p* = 0.043, *β* = −0.04), meaning (*p* = 0.011, *β* = −0.05), accomplishment (*p* = 0.02, β = −0.05), and health scores (*p* = 0.019, *β* = 0.05). Negative *β* values indicated that older participants tended to have lower scores in the overall well-being score and all six PERMAH areas.

**Table 6:** Demographic variables as regression coefficients of PERMAH and overall well-being.

	O		P		E		R		M		A		H	
Predictors	*β*	*p*	*β*	*p*	*β*	*p*	*β*	*p*	*β*	*p*	*β*	*p*	*β*	*p*
Age	**−0.05**	**0.02**	**−0.04**	**0.043**	−0.03	0.164	−0.02	0.268	**−0.05**	**0.011**	**−0.05**	**0.020**	**−0.05**	**0.019**
Sex	−0.03	0.46	−0.01	0.848	**−0.12**	**0.009**	0.04	0.409	−0.08	0.053	0.02	0.703	−0.07	0.085
Course cluster														
2 vs. 1	0.12	0.137	0.11	0.174	0.16	0.080	0.06	0.514	0.07	0.418	0.12	0.145	0.12	0.141
3 vs. 1	0.01	0.907	0.02	0.787	0.03	0.654	0.07	0.230	−0.09	0.119	−0.01	0.908	0.01	0.926
4 vs. 1	−0.06	0.408	−0.10	0.182	0.01	0.863	−0.03	0.733	−0.11	0.149	−0.00	0.966	**−0.15**	**0.040**
CMB														
B vs. A	−0.08	0.33	−0.13	0.130	−0.08	0.360	0.03	0.762	−0.07	0.393	−0.05	0.535	**−0.27**	**0.001**
C vs. A	−0.03	0.72	−0.13	0.113	0.07	0.442	0.08	0.380	−0.01	0.891	−0.09	0.288	**−0.18**	**0.022**
D vs. A	−0.10	0.219	**−0.21**	**0.013**	−0.01	0.902	0.04	0.632	−0.09	0.305	−0.12	0.170	**−0.20**	**0.016**
E vs. A	−0.08	0.325	−0.17	0.053	0.00	0.980	0.07	0.451	−0.12	0.188	−0.10	0.247	**−0.26**	**0.002**
F vs. A	−0.10	0.291	**−0.23**	**0.012**	0.04	0.689	−0.01	0.921	−0.09	0.327	−0.05	0.633	−0.14	0.140
G vs. A	−0.10	0.279	**−0.18**	**0.049**	0.08	0.433	0.01	0.938	−0.14	0.150	−0.11	0.284	**−0.26**	**0.006**

O, overall well-being; P, positive emotion; E, engagement; R, relationships; M, meaning; A, accomplishment; H, health; CMB, combined monthly income categories. Cluster 1, Arts and Letters; Cluster 2, Management and Economics; Cluster 3, Science and Technology; Cluster 4, Social Sciences and Law; CMB, Combined Monthly Income Categories; A, P0-P12,000; B, P13,000-P24,000; C, P25,000-P48,000; D, P49,000-P84,000; E, P85,000-P132,00; F, P133,000-P228,000; G, P229,000 and above; β, standardized beta; p, probability value. A higher absolute value of β indicates a stronger predictive relationship between the predictor and outcome variables. Positive values of β indicate a positive relationship, while negative values indicate a negative relationship. Bolded values indicate statistically significant relationships (*p* < 0.05).

Except for engagement (*p* = 0.009, *β* = −0.12), sex did not significantly explain the overall well-being and dimension scores in the presence of other predictors in the model. On average, it was found that females scored significantly lower in engagement than males, accounting for other independent variables in the model. The undergraduate course clusters did not significantly contribute to explaining the scores in the overall score and the areas except for health (*p* = 0.040, *β* = −0.15). The health scores of the students taking courses in the Social Sciences and Law cluster appeared significantly lower than those in the Arts and Letters cluster.

The combined monthly household income had no significant association with overall well-being, engagement, relationships, meaning, or achievement scores. For positive emotions and health, students with higher monthly incomes scored lower than those with lower incomes. Specifically, the combined household income brackets of ₱49,000 - ₱84,000 (*p* = 0.013, *β* = −0.21), ₱133,000 - ₱228,000 (*p* = 0.012, β = −0.23), and ₱229,000 and above (*p* = 0.049, β = −0.18) scored significantly lower in positive emotions than those with the lowest income bracket, ₱0-P12,000. Students in all income brackets except for ₱133,000–₱228,000 had *significantly* lower health scores than those in the lowest income bracket, with the ₱13,000–₱24,000 bracket holding other variables constant.

### Levels of physical activity as predictors of PERMAH

Table 7 summarizes the results of the seven multiple regression models focusing on PA levels as regression coefficients of PERMAH and overall well-being. These results demonstrate that the IPAQ category of participants contributed significantly to their overall well-being scores, according to the regression analysis employing the PERMAH profiler. Specifically, participants classified as engaging in health-enhancing PA (*p* < 0.001, *β* = 0.29) and those categorized as moderately active (*p* = 0.002, *β* = 0.16) exhibited significantly higher overall well-being scores than the insufficiently active students, with all other covariates being held constant.

**Table 7:** Levels of physical activity as regression coefficients of PERMAH and overall well-being.

	O		P		E		R		M		A		H	
Predictors	*β*	*p*	*β*	*p*	*β*	*p*	*β*	*p*	*β*	*p*	*β*	*p*	*β*	*p*
MA vs. IA	**0.16**	**0.002**	**0.20**	**<0.001**	0.08	0.134	**0.13**	**0.022**	**0.15**	**0.005**	0.06	0.248	0.08	0.117
HEPAA vs. IA	**0.29**	**<0.001**	**0.29**	**<0.001**	**0.21**	**0.002**	**0.16**	**0.015**	**0.30**	**<0.001**	**0.22**	**<0.001**	**0.17**	**0.007**

MA, moderately active; IA, insufficiently active; HEPA, health-enhancing physical activity; O, overall well-being; P, positive emotion; E, engagement; R, relationships; M, meaning; A, accomplishment; H, health; β, standardized beta; p, probability value. A higher absolute value of *β* indicates a stronger predictive relationship between the predictor and outcome variables. Positive values of *β* indicate a positive relationship, while negative values indicate a negative relationship. Bolded values indicate statistically significant relationships (*p* < 0.05).

Furthermore, students engaging in health-enhancing PA scored significantly higher in all PERMAH areas than those who were insufficiently active, fixing other covariates in this model. On the other hand, students classified as moderately active scored significantly higher in most PERMAH areas except for engagement and achievement than their insufficiently active counterparts, holding all other covariables constant.

### Dimensions of positive health behaviors as predictors of PERMAH

Table 8 summarizes results from seven multiple regression models focused on positive health behaviors as regression coefficients of PERMAH and overall well-being. The analysis showed that overall well-being, positive emotions, engagement, meaning, accomplishment, and health scores are positively associated with nutrition (*p* < 0.001, *β* = 0.07), PA (*p* < 0.001, *β* = 0.010), relaxation (*p* < 0.001, *β* = 0.40), and preventive behavior (*p* < 0.002, *β* = 0.16) after controlling for other relevant variables. Nutrition and PA were not significant predictors of relationship scores. No significant association was found between nutrition and engagement scores.

**Table 8:** Dimensions of positive health behaviors as regression coefficients of PERMAH and overall well-being.

	O		P		E		R		M		A		H	
Predictors	*β*	*p*	*β*	*p*	*β*	*p*	*β*	*p*	*β*	*p*	*β*	*p*	*β*	*p*
Nutrition	**0.07**	**0.001**	**0.07**	**0.002**	−0.01	0.845	0.03	0.207	**0.11**	**<0.001**	**0.08**	**<0.001**	**0.09**	**<0.001**
PA	**0.10**	**<0.001**	**0.07**	**0.002**	**0.08**	**0.001**	0.03	0.201	**0.09**	**<0.001**	**0.15**	**<0.001**	**0.25**	**<0.001**
Relaxation	**0.40**	**<0.001**	**0.042**	**<0.001**	**0.27**	**<0.001**	**0.29**	**<0.001**	**0.34**	**<0.001**	**0.29**	**<0.001**	**0.32**	**<0.001**
Preventive Behavior	**0.13**	**<0.001**	**0.07**	**0.003**	**0.12**	**<0.001**	**0.17**	**<0.001**	**0.09**	**<0.001**	**0.12**	**<0.001**	**0.05**	**0.024**

O, overall well-being; P, positive emotion; E, engagement; R, relationships; M, meaning; A, accomplishment; H, health. β, standardized beta; p, probability value. A higher absolute value of β indicates a stronger predictive relationship between the predictor and outcome variables. Positive values of β indicate a positive relationship, while negative values indicate a negative relationship. Bolded values indicate statistically significant relationships (*p* < 0.05).

With all four dimensions of positive health behaviors significantly predicting overall well-being and most PERMAH areas, the results imply that fostering healthy behaviors can positively impact well-being. These behaviors include eating breakfast, maintaining balanced nutrition, engaging in regular PA, limiting screen time, practicing relaxation techniques, getting adequate sleep, managing stress, fostering positive thinking, seeking help when needed, and spending time with family and friends. Additionally, encouraging preventive behaviors such as sun protection, dental care, health monitoring, vaccinations, and medical check-ups may further support overall well-being.

## Discussion

The current study looks into how sociodemographic factors, levels of PA, and health behaviors predict the well-being of Filipino undergraduate students. Results revealed that older students have lower well-being levels than their younger counterparts. Female students reported lower engagement scores compared to males. Those enrolled in social science and law programs exhibited lower health scores than those in the Arts and Letters cluster. Higher household income was linked to decreased positive emotions and health in the current sample. Participants who engaged in health-enhancing or moderate PA had significantly higher overall well-being scores and scores in most PERMAH areas than insufficiently active students. Nutrition, PA, relaxation, and preventive behaviors were positively associated with overall well-being and most PERMAH areas, with some exceptions for nutrition and PA concerning relationship and engagement scores.

### Age and well-being

The finding that older participants tended to have lower well-being scores aligns with previous research on student populations. Research reveals that subjective well-being weakens around age 10, reaches its lowest point in midlife, and strengthens after age 50 (Blanchflower, 2021). Similar findings have been reported in non-WEIRD settings like China, India, and Latin America, where older students acknowledge reduced well-being due to the compounded pressures of academic performance, financial obligations, and career uncertainty (Wang and Sheikh-Khalil, 2014). For instance, China espouses the concept of “face” (*mianzi*) with societal expectations for success, placing significant stress on students nearing graduation and contributing to burnout and declining engagement (Liu et al., 2024; Medinskaya, 2015). In many non-WEIRD countries, students from low- and middle-income families often shoulder economic burdens for their households, particularly as they approach graduation. These pressures reduce their mental well-being and ability to focus on academics, as seen in studies from Bangladesh (Muid et al., 2024) and Kenya (Kathuri-Ogola and Kabaria-Muriithi, 2024).

First-year college students typically exhibit high levels of enthusiasm and engagement as they are still adjusting to college life. However, by their later years in college, students report experiencing burnout, academic fatigue, and stress, leading to lower engagement and well-being (Schaufeli et al., 2002; Shankland et al., 2019). Older students will likely be in their final university years, juggling academic requirements, internships, research, and thesis work, leading to higher stress and lower well-being. The rigid structure of Philippine higher education, with heavy coursework and thesis requirements, often results in academic disengagement in the upper years.

Older Filipino students tend to have lower well-being scores due to socioeconomic, psychological, and academic factors. Socioeconomic levels significantly impact well-being. Students from lower socioeconomic backgrounds often experience higher stress and lower well-being due to financial constraints and related pressures (Villarino et al., 2023). The pressure of academic responsibilities, especially for those nearing graduation, can be overwhelming. This stress is often higher among older students who may also be confronting career-related decisions (Regalado, 2024). These issues could be exacerbated by cultural factors, including high expectations of academic, professional, and financial success placed on older students by their families and society, which can compound their stress and negatively impact their well-being (Alejandria et al., 2023).

### Culture and socioeconomic factors

The Filipino culture of “*utang na loob*” (debt of gratitude) implicitly pressures students to reciprocate the support and sacrifice of their parents with academic success to ensure their contribution to supporting their families after graduation (Enriquez, 1994). Many older students do not wait until graduation to work as they accept part-time jobs to support their families or earn their keep to avoid financially burdening their families, affecting their mental health and academic engagement. Older students also face increasing pressure to secure employment, especially in a highly competitive job market where youth unemployment remains high (Asian Development Bank, 2021). Job market uncertainty, financial instability, and family expectations contribute to lower life satisfaction and well-being among students nearing graduation. As they near the completion of their degree programs, many may struggle with self-doubt regarding career choices, which can cause anxiety and disengagement from academic life (Chamandy and Gaudreau, 2019; Wong and Kaur, 2017; Zhang et al., 2021). Moreover, as students mature and advance in the university, their social circles shrink due to varying academic schedules, internships, and off-campus responsibilities. Losing peer support and social engagement leads to increased isolation and declining engagement in school activities (Buote et al., 2007; Shalaby and Agyapong, 2020).

### Gender and academic engagement

This study highlights a notable disparity in academic engagement, with female students exhibiting lower engagement levels than their male counterparts. The finding aligns with existing literature, which attributes such differences to structural and sociocultural factors. One explanation for this disparity lies in the misalignment between female students’ gender identity and the masculine stereotyping of specific academic fields, particularly in science, technology, engineering, and mathematics (STEM) disciplines. Studies suggest that this mismatch can lead to reduced engagement and higher risks of school burnout among female students (Kessels, 2023; Lawson, 2021). Moreover, the perception of academic effort as a feminine trait may inadvertently devalue girls’ academic accomplishments and contribute to their underrepresentation in traditionally male-dominated fields such as STEM and related disciplines (Kessels, 2023).

This stereotype reinforces the notion that girls who excel academically do so through effort rather than innate ability, which can lead to devaluing their accomplishments (Kessels, 2023). Leslie et al. (2015) found that fields emphasizing innate talents—such as physics, mathematics, and philosophy—have lower female representation, as these fields implicitly reward perceived “genius” rather than effort. The cultural bias associating brilliance with masculinity creates a barrier for women, discouraging them from pursuing STEM careers and reinforcing the perception that they do not “naturally belong” in these disciplines (Nosek et al., 2002). This gendered perception can influence how parents, teachers, peers, and even students themselves interpret academic success (Miller et al., 2024). Bian et al. (2017) demonstrated that as early as 6 years old, girls begin to internalize the belief that they are less likely than boys to be “really, really smart.” When young girls experience consistent feedback that their success is due to effort rather than ability, it can shape their self-perception, leading them to undervalue their achievements and develop lower academic self-confidence (Eccles, 2011).

Furthermore, the perception of effort as feminine can make academic achievement a social liability for girls, particularly in adolescence. Kessels (2023) notes that girls who demonstrate strong academic performance in male-dominated fields may experience social backlash because excelling in subjects like math and science contradicts gender norms. This scenario can contribute to their withdrawal from STEM-related activities and reduced aspirations for high-status careers in these fields. Because STEM disciplines often prioritize perceived natural talent over hard work, women who have been socialized to associate their achievements with effort rather than ability may feel discouraged from pursuing careers in these fields (Cech, 2022). When success is framed as requiring brilliance rather than dedication, and brilliance is seen as a masculine trait, it can reinforce gender disparities in career paths (Leslie et al., 2015).

Gendered patterns of academic behavior extend beyond STEM. Guo et al. (2024) found that female Chinese students are less likely to engage in higher-order learning behaviors, resource-intensive educational practices, and student-faculty interactions, which are crucial for academic engagement and success. Similarly, in male-dominated majors, female students report lower levels of daily engagement in courses than their peers, which can influence the quality of their social and academic experiences (Lawson, 2021). Additionally, female students tend to perceive their academic load as more demanding, especially when exposed to gender stereotypes. This heightened perception of academic pressure can negatively affect their engagement levels (Maloshonok et al., 2022).

Beyond academic stereotypes, sociocultural factors in the Philippines may also significantly shape engagement levels. As a traditionally patriarchal culture, the Philippines upholds gender norms that emphasize passivity, caretaking, and modesty for women, while men are encouraged to be assertive and independent (Medina and Guzman, 2015). These societal expectations can extend to educational settings, where male students feel more encouraged to actively participate in class discussions, extracurricular activities, and sport. In contrast, female students may be socialized to adopt a more reserved stance, potentially limiting their classroom engagement and broader involvement, particularly in the presence of male classmates in co-educational settings.

Environmental factors further contribute to these disparities. In urban centers such as Manila, Cebu, and Davao, safety concerns—including harassment and gender-based violence—can restrict women’s freedom of movement (Foundation for Media Alternatives, 2023; Rivera, 2022; World Bank Group, 2020). As a result, female students may be less likely to participate in after-school or evening extracurricular activities, student organizations, or leadership opportunities, further reducing their engagement levels and overall well-being.

Gendered psychological coping mechanisms may also play a role in shaping engagement levels. Research suggests that women are more likely to internalize stress, leading to higher rates of anxiety and depression, which can negatively impact motivation and academic involvement (Graves et al., 2021). Conversely, men are more likely to engage in active coping mechanisms, such as participating in sport or socializing, which may contribute to higher levels of engagement in both academic and extracurricular activities. Traditional masculinity norms further encourage assertiveness and self-expression, fostering greater engagement in classroom discussions and group activities.

Gender disparities in academic engagement are not unique to the Philippines. Studies in non-WEIRD contexts, such as South Asia, Africa, and Latin America, report similar patterns of lower well-being among women, often due to gender inequities, societal expectations, and limited opportunities. For example, Singh and Raina (2020) found that female students reported lower engagement and self-efficacy, primarily due to gendered academic pressures and restrictive cultural norms. In sub-Saharan Africa, women often face additional burdens from household responsibilities and limited access to education and resources, negatively affecting their well-being (UNESCO, 2022). Similarly, in India and the Middle East, women’s restricted mobility and safety concerns contribute to reduced participation in outdoor activities and sport, which affects physical and mental well-being (Koranga et al., 2024; Saeed et al., 2023).

In contrast, WEIRD societies, such as the United States and Europe, tend to exhibit smaller gender disparities in engagement, partly due to more egalitarian educational systems and policies promoting gender equity in leadership and extracurricular activities (Bleidorn et al., 2016). For instance, female students in Scandinavian countries often outperform males in engagement and leadership participation due to pro-women policies and cultural attitudes toward equality (Organisation for Economic Co-operation and Development, 2019). Additionally, women in WEIRD contexts may be more likely to engage in active coping mechanisms, such as seeking social support or therapy, compared to women in non-WEIRD societies, where cultural stigmas around mental health and emotional expression often persist (Folberg, 2020; Lin et al., 2024).

The gender disparities observed in this study underscore the need for targeted institutional efforts to foster more inclusive and supportive learning environments for female students. Universities should implement policies that ensure equal access to leadership opportunities, extracurricular activities, and mental health support. Addressing restrictive gender norms, promoting safer campus environments, and encouraging active engagement among female students can help mitigate the sociocultural barriers that limit their academic participation.

### Income and well-being

One of the study’s unexpected findings is the inverse relationship between income and well-being, particularly regarding positive emotions and health scores. Students from higher-income brackets reported lower well-being than those from lower-income groups. This result challenges traditional views that suggest higher income leads to better well-being due to increased access to resources and improved living conditions (Asebedo et al., 2022; Diener and Biswas-Diener, 2002; Killingsworth et al., 2023) and lower income with poorer health outcomes (Bor et al., 2017; Mullahy et al., 2018; Truesdale and Jencks, 2016). However, the Easterlin Paradox (Easterlin, 1974) posits that income increases well-being up to a certain point, beyond which additional financial gains yield diminishing returns to happiness. Similarly, the concept of hedonic adaptation (Diener et al., 2006) explains that people gradually adjust to higher income levels, reducing well-being anchored on financial security over time.

A probable explanation for this phenomenon hinges on Filipino families’ cultural valuation of academic achievement. Students from higher-income backgrounds may experience heightened academic or social pressures due to family expectations and the provision of resources. Research indicates that Asian immigrant parents, including Filipino families, often hold high academic and professional expectations for their children (Mun and Hertzog, 2019; Naumann et al., 2012; Yii, 2024). The concept of filial piety, which emphasizes making parents proud, can intensify these pressures as children strive to fulfil their familial obligations (Cherng and Liu, 2017; Mun and Hertzog, 2019). Consequently, students from affluent backgrounds may experience more significant stress, sacrificing rest, recreation, and sleep to meet these expectations. This lifestyle may reduce engagement in stress-relieving activities, ultimately leading to lower well-being scores despite financial stability. Moreover, existing research reveals that students from wealthier backgrounds often experience higher rates of anxiety and perfectionism due to elevated parental and societal expectations (Becker and Luthar, 2002; Liu et al., 2018; Luthar and Becker, 2002). These psychological stressors may outweigh financial security benefits, contributing to lower positive emotions and overall well-being among higher-income students. However, the results of the current study should be interpreted with caution, given that normality tests violated the regression model’s assumption. Further research is necessary to confirm these results and investigate the underlying causes of these unexpected patterns in the data.

### Positive health behaviors and health-enhancing physical activity

For over three decades, the relationship between mental health and PA has been extensively studied (Sabe et al., 2022). Research consistently highlights the importance of positive health behaviors as strong predictors of overall well-being (Chilver et al., 2023; Wickham et al., 2020). However, in the present study, participants reported low PA levels on the PHBS (*M* = 4.86, SD = 2.71). The low standard deviation indicates that most scores are clustered around the mean, with 23% of participants classified as insufficiently active, according to the IPAQ. Despite these low activity levels, this study found that engaging in health-enhancing PA significantly predicts all elements of the PERMAH model, encompassing overall well-being and health. Walking, cycling, performing sport, and vigorous recreation positively impact physical and mental health. This study outcome is supported by existing literature (Lawton et al., 2017; Rector et al., 2019), reinforcing the positive relationship between physical exercise and well-being across diverse populations. The findings suggest that intervention programs to improve student well-being should focus on increasing PA levels.

The findings also highlight the significance of health-enhancing PA as a robust predictor of all PERMAH elements, including well-being and overall health. Notably, the data were collected 8 months after the national pandemic restrictions on PA and social interactions were lifted; thus, the low PA scores of the participants are concerning. According to IPAQ, only 27% of the participants engage in health-enhancing PA. To address this, future interventions can harness PE classes that offer various courses in dance, sport, exercise, leisure, martial arts, and traditional Philippine games across the university system. In the study locale, students are required to take four semesters of PE classes, with the option to take additional courses, offering a sustained opportunity for PA throughout their college years, which can support their well-being.

While there is limited research on post-pandemic PA (PA) and health behaviors, a study by McCarthy et al. (2021) found that PA levels among their participants increased immediately after COVID-19 lockdowns were lifted in the United Kingdom. In contrast, the current study, conducted 8 months after pandemic restrictions were eased in the Philippines, reported that PA levels and adherence to health behaviors remain low among Filipino tertiary students. The persistent inactivity and low adherence to health behaviors among the study demographics are alarming, particularly in light of recent research that highlights the positive impact of PA on mental health during and after the pandemic (Li et al., 2022). Other studies (Ai et al., 2021; Marconcin et al., 2022) have demonstrated that PA can mitigate the adverse mental health effects associated with the pandemic. Despite the easing of restrictions, the persistence of a sedentary lifestyle among this cohort underscores the need for further investigation into the factors influencing post-pandemic PA behaviors and the development of effective interventions to promote PA in the “new normal.”

### Nutrition, relaxation and preventive behaviors

The study also sheds light on the varying adherence to nutrition-related behaviors, with participants showing a strong commitment to regular meal patterns and vegetable consumption. These results support Grajek et al. (2022), who found that a diet rich in fruits, vegetables, lean meats, and whole grains supports optimal bodily function. The moderate adherence observed in this study to regular meals, especially nutrition-rich breakfasts, resonates with research showing that consuming a nutritious breakfast is associated with lower levels of depression and stress, higher happiness scores, and better quality of life (Ferrer-Cascales et al., 2018; Lundqvist et al., 2019). Additionally, Beilharz et al. (2015) emphasize the negative impact of foods high in processed sugar and saturated fat on cognition, particularly memory functions and mood.

The study also revealed the suboptimal dairy intake, particularly of fermented products like kefir and yoghurt, which contrasts with Zidan et al. (2024). They assert that the loss of beneficial gut bacteria can contribute to mood disorders, and their review of 13 studies concluded that consuming probiotic supplements and fermented foods such as kefir and yoghurt can improve mood in middle-aged and older women. The term “psychobiotic” is used (Gobel and Doğan, 2023; Ross, 2023; Zidan et al., 2024) to refer to probiotics or prebiotics that, when consumed appropriately, positively influence mental health by regulating various markers like chemokines, cytokines, neurotransmitters, or neurotrophins levels that circulate in the blood (Heidarzadeh-Rad et al., 2020).

Engagement in relaxation practices (Rooks et al., 2017) and ensuring adequate sleep enhance well-being (Ong et al., 2017) were also identified as key factors in enhancing well-being. Most participants reported frequent engagement in relaxation practices, though there was considerable variability in sleep duration and regularity. Many reported only occasionally getting 6–7 h of sleep and rarely maintaining a regular sleep schedule. Relaxation techniques such as progressive muscle relaxation and deep-breathing exercises have been shown to yield significant benefits. Toussaint et al. (2021) suggest that participating in relaxation efforts can lower stress, while Hamdani et al. (2022) show their efficacy in alleviating anxiety, sadness, and distress symptoms.

Effectively coping with stress is crucial for overall well-being, particularly among young adults. Akhtar and Kroener-Herwig (2019) assert the importance of developing coping mechanisms, particularly those focused on problem-solving and emotional regulation. Chen (2016) advocates for coping strategies like seeking social support and practicing relaxation techniques, while Maia de Carvalho and Vale-Dias (2013) stress the need for tailored coping skills training programs to address stressors, such as academic demands and career transitions.

The findings on positive thinking corroborate existing literature. Positive thinking has been found to advance psychological well-being (Alkhatib, 2020) and improve stress management (Brown, 2021; Tolukan et al., 2024). Davies (2009) extends this concept by introducing hope—a positive future outlook—which has been associated with improved well-being (Gallagher et al., 2021; Murphy, 2023; Pleeging et al., 2021; Yıldırım and Arslan, 2022). Ekman et al. (2022) explore broader cognitive habits, including optimism, stress management, autonomous decision-making, seeking variety and challenges, social connection, continuous learning, repetition, and healthy practices like eating, exercise, and restful sleep.

Goodwin et al. (2016) found that informal help-seeking from friends positively correlates with well-being, while Ratnayake and Hyde (2019) highlight the need for mental health literacy to enable individuals to identify psychological distress and seek appropriate support needed. Additionally, as Brajša-Žganec et al. (2011) identified, active socializing was also found to predict well-being. This study affirms that engaging in preventive health behaviors, such as dental care (Alsumait et al., 2015) and taking vitamins and minerals as supplements (Kumar et al., 2020; Maggini et al., 2021), is strongly associated with well-being. These behaviors support physical health and prevent diseases or injuries that could negatively impact well-being.

### Recommendations

Given these findings, a multi-faceted approach to improving the well-being of tertiary students is recommended, focusing on the interplay between physical and psychological health. Universities should proactively encourage and facilitate PA by providing access to sport facilities, fitness centers, and exercise classes. Incorporating PA into the curriculum through movement-based activities across various courses can benefit well-being in the school setting. For example, teachers could introduce PA breaks during class sessions to reduce prolonged sitting behavior and sedentary behavior, which is common among young people (Feiler, 2019). Universities can also organize programs centered on physical health literacy, amplifying the interconnectedness between physical and mental health. These endeavors seek to inform and motivate students to embrace positive lifestyle choices through participation in activities such as nutrition workshops, sessions on sleep hygiene, and stress reduction practices.

One example of a successful intervention is a program that integrated the PERMAH framework into PE classes in a Philippine university (Campoamor-Olegario et al., 2024). The program included mindfulness walks, gratitude exercises, and goal setting to promote positive emotions, engagement, relationships, meaning, accomplishment, and health. Students who participated in the program reported finding greater meaning in their PE classes and feeling more comfortable and confident in their interactions with family and friends.

In addition, Diaz et al. (2024) reported the positive impact of a mindfulness-based PE course on student well-being in the same Philippine university. A course called Mindfulness for Health and Well-being aimed to teach mental health skills and promote prosocial behavior among college students through mindfulness practices and cognitive behavioral therapy exercises. Similarly, Morgan and Simmons (2021) developed an online positive education program based on the PERMA framework to promote well-being in university students. Their program focused on building positive emotions, engagement, relationships, meaning, and accomplishment, improving participant well-being outcomes. These studies provide further evidence for the effectiveness of incorporating positive psychology interventions within PE to enhance student well-being.

Schools can also enhance dining options by offering healthier food selections in cafeterias. Additionally, students can be encouraged to develop physical well-being and uphold mental health by integrating preventive health practices into their daily routines. Educational institutions can provide students and faculty members with mental health resources and support services. Recognizing the positive impact of these interventions on well-being, policymakers, and decision-makers can advocate for a holistic approach to health promotion and disease prevention, thereby fostering the cultivation of thriving communities.

While the study generates valuable insights into the predictive relationship between PA and positive health behaviors in overall well-being, further investigations are recommended to fully comprehend the complex interplay of variables examined. Specifically, future studies employing longitudinal designs are needed to establish causality and determine the long-term effects of PA and positive health behaviors on student well-being. The long-term well-being trajectories of specific subgroups, such as female students, older students, or those from low-income households, can also be explored. Expanding research beyond tertiary education to include school students could also clarify how various health behaviors are established over time. Comparative studies examining the behaviors and well-being of individuals who do not pursue higher education versus those in tertiary institutions could further enrich the discourse.

Future research could identify the particular types of PA and health behaviors that most effectively enhance college students’ well-being. Moreover, qualitative approaches may also be employed to explore underlying mechanisms in the relationship between positive health behaviors and student well-being. These could include interviews to explore the interaction of gender and socio-economic factors in health and fitness. By broadening the scope of inquiry and incorporating diverse student populations, researchers can contribute to a more comprehensive understanding of well-being across different educational and social contexts. Additionally, incorporating qualitative methods such as focus groups or interviews could offer deeper insights into the reasons behind these trends. Including more diverse student populations in future research would also enhance the validity of the findings and provide a more comprehensive understanding of well-being in tertiary education.

### Limitations

While the study provides valuable insights into the relationship between positive health behaviors, physical activities, and well-being among Filipino tertiary students, it is crucial to consider that it is not without limitations. One stems from self-report data, which can be subject to recall, social desirability, and interpretation biases. Participants might have inadvertently overestimated their PA levels or adherence to healthy habits. Future researchers may use more objective tools like accelerometers or dietary records to complement self-report data.

Though the sampling approach was designed to include diverse participants, it focused primarily on students enrolled in Physical Education courses. This focus may have inadvertently excluded certain groups, introducing a degree of sampling bias. Broader sampling techniques would help future research capture a more representative snapshot of the overall student population.

Lastly, the study’s context is specific to Filipino tertiary students, which may limit the findings’ generalizability to other cultural settings. Similar studies across varied cultural settings may be needed to ascertain cross-cultural validity.

These limitations underscore the need for caution in generalizing the findings and emphasize the importance of future investigations that address these limitations to provide a clearer picture of the complex relationship between positive health behaviors, PA, and well-being in student populations.

## Conclusion

This study contributes to the literature on physical and psychological health by reinforcing the predictive role of PA and positive health behaviors in overall well-being, including positive emotions, engagement, meaning, accomplishment, and health metrics. The findings strongly support the hypothesis that PA levels and dimensions of positive health behaviors significantly predict well-being among Filipino tertiary students. Those who engage in positive health behaviors and maintain moderate PA levels consistently score higher across all six well-being domains than their inactive peers.

A novel contribution of this research is its meticulous examination of how different levels and types of PA, along with other healthy behaviors like good nutrition, regular relaxation, and preventive measures, distinctly influence various aspects of well-being. This analysis addresses a critical gap in the literature by identifying specific behavioral trends linked to improved well-being outcomes.

Additionally, the study confirms that individuals who engage in health-promoting activities and maintain regular activity consistently exhibit higher well-being scores across all six domains than those who are inactive. It highlights that sound nutrition, PA, relaxation, and preventive behaviors favorably predict overall well-being, meaning, and accomplishment. Notably, preventive and relaxing behaviors were the only ones significantly related to the relational components of well-being, suggesting a complex connection between health behaviors and the social dimensions of well-being that warrants further investigation.

Understanding the differential impacts of various activities on relationships requires a more in-depth investigation to determine which activities provide the greatest benefits. Longitudinal studies are essential to assess the sustainability of these effects over time. Expanding this research to diverse settings will allow for broader validation of the current findings and help identify specific cohorts that may benefit differently from these activities. Finally, given these results, the study advocates for targeted public health initiatives that prioritize disseminating practical information on the benefits of PA for well-being, promoting regular PA through online and onsite platforms, and integrating relaxation techniques and preventive health practices into daily routines.

## Data Availability

The datasets presented in this article are not readily available because the original consent obtained from participants do not include provisions for sharing the data beyond the research team. Moreover, The IRB that approved the study have imposed restrictions on data sharing to protect the participants. Requests to access the datasets should be directed to Desiderio Camitan IV, dscamitan@up.edu.ph.
